# Mouse SIRT3 Attenuates Hypertrophy-Related Lipid Accumulation in the Heart through the Deacetylation of LCAD

**DOI:** 10.1371/journal.pone.0118909

**Published:** 2015-03-06

**Authors:** Tongshuai Chen, Junni Liu, Na Li, Shujian Wang, Hui Liu, Jingyuan Li, Yun Zhang, Peili Bu

**Affiliations:** 1 Department of Cardiology, Qilu Hospital, Shandong University, Jinan, Shandong, China; 2 Key Laboratory of Cardiovascular Remodeling and Function Research, Chinese Ministry of Education and Chinese Ministry of Public Health, Qilu Hospital, Shandong University, Jinan, Shandong, China; Texas A& M University Health Science Center, UNITED STATES

## Abstract

Cardiac hypertrophy is an adaptive response to pressure, volume stress, and loss of contractile mass from prior infarction. Metabolic changes in cardiac hypertrophy include suppression of fatty acid oxidation and enhancement of glucose utilization, which could result in lipid accumulation in the heart. SIRT3, a mitochondrial NAD+-dependent deacetylase, has been demonstrated to play a crucial role in controlling the acetylation status of many enzymes participating in energy metabolism. However, the role of SIRT3 in the pathogenesis of hypertrophy-related lipid accumulation remains unclear. In this study, hypertrophy-related lipid accumulation was investigated using a mouse cardiac hypertrophy model induced by transverse aortic constriction (TAC). We showed that mice developed heart failure six weeks after TAC. Furthermore, abnormal lipid accumulation and decreased palmitate oxidation rates were observed in the hypertrophic hearts, and these changes were particularly significant in SIRT3-KO mice. We also demonstrated that the short form of SIRT3 was downregulated in wild-type (WT) hypertrophic hearts and that this change was accompanied by a higher acetylation level of long-chain acyl CoA dehydrogenase (LCAD), which is a key enzyme participating in fatty acid oxidation. In addition, SIRT3 may play an essential role in attenuating lipid accumulation in the heart through the deacetylation of LCAD.

## Introduction

Cardiac hypertrophy is an adaptive response that is accompanied by many forms of heart disease, including ischemic disease, hypertension, heart failure, and valvular disease [1–3]. In these types of cardiac pathology, pressure overload-induced concentric hypertrophy is believed to have a compensatory function by diminishing wall stress and oxygen consumption. However, ventricular hypertrophy is also associated with significantly increased risk of heart failure and malignant arrhythmia [4,5]. Thus, it is important to suppress hypertrophy without provoking circulatory insufficiency.

To accomplish this goal, it is critical to elucidate the mechanisms underlying the maladaptive features of hypertrophy. Of the many maladaptive features of hypertrophy, energy metabolism, which reverses the oxidation of long-chain fatty acid to increased glucose utilization, makes a large difference in the process of hypertrophy [6]. On the one hand, it decreases oxygen consumption per mole of ATP generated, which could help reduce cellular reactive oxygen species (ROS), and on the other hand, maladaptive features exist, including increased lipid accumulation in the heart stemming from chronically impaired oxidation of fatty acid, lactic acid accumulation, and diminished maximal ATP generation from glycolysis. However, the molecular mechanism through which lipid accumulates in the heart during hypertrophy remains unknown.

Recent studies have found that sirtuins are critical regulators of many cellular processes, including insulin secretion, the cell cycle, and apoptosis [7–9]. It has been proven that sirtuins are novel therapeutics that inhibit metabolic disorders and combat associated diseases [10,11]. Of the seven sirtuin analogues, SIRT3 is the only member whose increased expression has been linked to the longevity of humans [12,13]. SIRT3 belongs to the class III of histone deacetylases, which require NAD^+^ as a cofactor for their deacetylation reaction. It has been proven that SIRT3 facilitates lipid, amino acid and carbohydrate metabolism. Long-chain acyl CoA dehydrogenase (LCAD), a key enzyme involved in fatty acid oxidation, has been identified as a target of SIRT3 [14–17]. Moreover, recent studies have demonstrated that SIRT3 also exhibits protection effects on cardiomyocytes by reducing ROS production [18–21]. However, the molecular basis of SIRT3-mediated protection on hypertrophy-related lipid accumulation remains unknown.

To explore the role of SIRT3 in hypertrophy-related lipid accumulation, we subjected WT mice and SIRT3-KO mice to transverse aortic constriction (TAC). The results show that the SIRT3-KO mice were inclined to develop lipid accumulation in the heart. In addition, the short form of SIRT3 was downregulated during hypertrophy in wild-type (WT) mice, and this effect was accompanied by a higher acetylation level of LCAD. The overexpression of SIRT3 in cardiomyocytes could attenuate lipid accumulation through the deacetylation of LCAD. Our findings suggest that SIRT3 may block heart hypertrophy by inhibiting lipid metabolism disorders and attenuate lipid accumulation in the heart mitochondria through the deacetylation of LCAD.

## Materials and Methods

### Ethics Statement

The animal experimental protocol complied with the Animal Management Rules of the Chinese Ministry of Health (Document No. 55, 2001) and was approved by the Animal Care and Use Committee of Shandong University. The SIRT3-KO mice were purchased from the Jackson Laboratories (USA). 129 Wild-type mice at 6–7 weeks of age were purchased from the Department of Laboratory Animal Science of Peking University and served as controls (Beijing, China). All animals were maintained at the key Laboratory of Cardiovascular Remodeling and Function Research at Qilu Hospital of Shandong University.

### Reagents

Phenylephrine (PE) was purchased from Sigma Aldrich (USA). Antibodies against acetyllysine, SIRT3 (28 kDa), LCAD, β-actin, Hsp70, 3-Flag, histone3, α-SMA, PGC-1α and PPARα were purchased from Cell Signaling Technology (UK) and SIRT3 (44 kDa) was purchased from Abgent (USA) and Abcam (USA).

### Animal protocol

Eight-week-old male mice (age-matched wild-type and Sirt3-KO mice with a body weight of 23±1.5 g) were subjected to constriction of the thoracic aorta as described previously [22]. The SIRT3-KO mice were born at a Mendelian ratio [23], and both WT and SIRT3-KO mice were littermates. The animals were anesthetized using sodium pentobarbital, and the aorta was isolated from the adjacent tissue and banded between the carotid arteries over a 27-gauge needle, which was immediately removed. The animals subjected to sham surgery underwent an identical procedure with the exception of band placement. The animals were sacrificed six weeks after surgery, and their hearts were removed and analyzed for the development of cardiac hypertrophy and lipid analyses.

### Cell culture, transfection and treatment

Rat cardiomyocytes (H9C2) were obtained from American Type Culture Collection (ATCC) and maintained in DMEM (Gibco) containing 10% FBS and penicillin/streptomycin (Invitrogen, Carlsbad, CA, USA) in a 5% CO_2_ humidified incubator at 37°C. For all of the adenovirus experiments, the viruses were used at a multiplicity of infection of 10. HEK 293 cells were grown in Dulbecco’s modified Eagle’s medium supplemented with penicillin-streptomycin and 10% fetal bovine serum (complete growth medium). The cells were transfected with appropriate plasmids using the Superfect transfection reagent (Qiagen) according to the manufacturer’s protocol. Rat cardiomyocytes (H9C2) were treated with PE (20 μM) for 48 h to induce hypertrophy *in vitro*.

### Histopathology and immunohistochemistry

The hearts were fixed in 4% paraformaldehyde, embedded in paraffin, and sectioned at 5-μm intervals. Hematoxylin and eosin staining and Masson’s trichrome staining were performed using standard procedures [24,25]. The collagen volume fraction was quantified blindly using quantitative morphometry with an automated image analysis system (Image-Pro Plus, Version 7.0, Media Cybernetics, Silver Spring, MD, USA). The cardiac collagen volume fraction was calculated as a ratio of (the sum of total interstitial collagen area) to (the sum of total connective tissue and muscle area) in the entire visual field of the section while excluding perivascular collagen, as reported previously. Measurements from 3 heart sections (8–10 fields per section) per rat were averaged for all parameters.

### Echocardiography of mice

The mice were anesthetized lightly using sodium pentobarbital. The animals were imaged in the left lateral decubitus position with a VisualSonics Vevo 770 machine using a 30 MHz high-frequency transducer. Images were captured from M-mode, two-dimensional (2-D), pulse-wave (PW) Doppler [26].

### Transmission electron microscopy

Left ventricular myocardial tissues with dimensions of approximately 0.5 mm × 1 mm × 1 mm from each group were fixed overnight with 2% glutaraldehyde, washed three times using 0.2 mol L^−1^phosphate buffer, re-fixed with 1% osmium tetraoxide, washed with 0.2 mol L^−1^phosphate buffer and dehydrated using an ethanol series. The samples were immersed in Epon812 resin acetone (1:1) for 30 min, and then embedded for convergence overnight at 70°C. The tissue was cut into pieces, and 50-nm-thick slices were generated using an ultra-microtome (LKB880, LKB Produkter AB, Bromma, Sweden). The embedded sections were stained using lead citrate and observed using a transmission electron microscope (TEM, H-7000FA, Hitachi, Tokyo, Japan).

### Triglyceride and cholesterol assays

The triglyceride (TG) and cholesterol contents in the heart extracted by a chloroform/methanol (2:1) mixture were determined using the TG Assay Kit (Biovision Inc. Mountain View, CA, USA) and a Cholesterol/Cholesteryl Ester Quantitation Kit (Biovision Inc.).

### Western blotting analysis

The proteins (50μg) were separated by SDS-PAGE and transferred onto a PVDF membrane using a wet transfer apparatus (Bio-Rad, Hercules, CA, USA). The membranes were blocked with 5% non-fat milk, incubated overnight at 4°C with the primary antibodies, and then incubated with the secondary antibodies labeled with horseradish peroxidase [27]. The protein bands were visualized through enhanced chemiluminescence (Millipore). The protein levels were detected using an ImageQuant LAS4000 chemiluminescence reader (GE, USA). The protein levels were analyzed by using the ImageJ software.

### Subcellular fractionation and immunoprecipitation

Subcellular protein fractions of mouse hearts were prepared using a ProteoExtract Subcellular Proteome Extraction kit (Calbiochem) according to the manufacturer’s protocol. The proteins were lysed in ice-cold LMIP buffer (1%n-dodecyl-D-maltoside, 0.5 mM EDTA, 150 mM NaCl, 10 mM nicotinamide, 1 mM trichostatin A, and 50 mM Tris-HCl, pH 7.4) containing a complete EDTA-free protease inhibitor cocktail (Roche). The immunoprecipitation was performed according to standard procedures. The immune complexes were washed four times in NP1 buffer (1% NP-40, 300 mM NaCl, 0.5 mM EDTA, and 50 mM Tris-HCl, pH 7.4).

### Real-time PCR analysis

The *sirt3*, *anf*, *β-mhc*, *lcad*, *cpt-1* and *β-actin* mRNA levels were quantified by SYBR Green Real-Time PCR (Takara) using a previously described protocol [28]. The genotyping for Sirt3-deficient mice was performed using the following primers: wild-type forward, 5′-CTT CTG CGG CTC TAT ACA CAG-3′; common, 5′-TGC AAC AAG GCT TTA TCT TCC-3′; mutant reverse, 5′-TAC TGA ATA TCA GTG GGA ACG-3′; ANF forward, 5′-TAA GCC CTT GTG GTG TGT CA-3′; and reverse, 5′-GCA AGA CCC CAC TAG ACC AC-3′; β-MHC forward, 5′-AAG GGC CTG AAT GAG GAG TA-3′; and reverse, 5′-AAA GGC TCC AGG TCT GAG G-3′; β-actin forward, 5′-CAA GAT CAT TGC TCC TCC TG-3′; and reverse, 5′-TCA TCG TAC TCC TGC TTG CT-3′; LCAD forward, 5′-AAG GAT TTA TTA AGG GCA AGA AGC-3′; and reverse, 5′-GGA AGC GGA GGC GGA GTC-3′; and CPT-1 forward, 5′-CGT CTT TTG GGA TCC ACG ATT-3′; and reverse, 5′-TTA AAC ATC CGC TCC CAC TGA GCG G-3′.

### Immunofluorescence

The cardiomyocytes were fixed for 15 min in 4% paraformaldehyde prepared in complete medium at 37°C in an incubator. Briefly, the cells were incubated with primary antibodies for α-SMA overnight at 4°C in a humid chamber and then incubated for 1 h with the appropriate secondary antibody. All of the microscopy and imaging analyses were performed in the digital confocal microscopy core facility.

### Blood analyses

After 4 h of fasting, the mice were anesthetized with isoflurane. We collected orbital blood and analyzed the serum to determine the triglyceride and cholesterol levels with a Bayer 1650 blood chemistry analyzer (Bayer, Tarrytown, NY, USA). The 4-h fasting blood glucose levels were measured with Accu-Chek (Roche Diagnostics, Nutley, NJ, USA) without anesthesia.

### Oil Red O staining and cholesterol and triglyceride measurements of cardiomyocytes

The lipid contents in cardiomyocytes were stained with Oil Red O. The cells were fixed in 10% formalin for 90 min. After washing thoroughly with distilled water, the cells were incubated with a working solution of Oil Red O for 3 h.

The cholesterol and triglyceride contents in the cells were measured quantitatively through enzymatic colorimetric assays using kits purchased from Wako (Richmond, VA, USA) according to the manufacturer’s protocols. The concentrations of cellular proteins in these cells were measured using a protein assay kit obtained from Bio-Rad (Hercules, CA, USA).

### Isolated Langendorff perfusion and palmitate oxidation rate

WT and SIRT3-KO mice were fasted overnight and terminally anaesthetized intraperitoneally with sodium pentobarbital (Euthatal, Merial, UK). The hearts were rapidly excised and placed in ice-cold modified Krebs-Henseleit (KH) buffer containing 11 mM glucose. The hearts were cannulated via the aorta and perfused in the Langendorff mode with KH buffer (gassed with 95% O2, 5%CO2) at 37°C and a constant perfusion pressure of 100 mmHg. After an initial 15-min stabilization period, the hearts were perfused with 250 ml of recirculating KH buffer containing 1 mM palmitate bound to 1.5% fatty acid-free bovine serum albumin (Sigma, USA) and 0.2 μCi ml^-1^ [9, 10−^3^H] palmitate for 1 h. Aliquots of recirculating buffer were collected every 8 min during the perfusion protocol, and the palmitate oxidation rates were determined. Briefly, ^3^H_2_O was separated from ^3^H-palmitate in the buffer samples through chloroform:methanol Folch extraction, and the upper aqueous phase was assessed for radioactivity. The steady-state palmitate oxidation rates were calculated from the linear increase in ^3^H_2_O, which was expressed as μmol × gram of whole heart wet weight (gww^−1^) min^−1^.

### Statistical analysis

The student’s two-tailed t-test was used to determine the significance of the differences between the treatment and control values. The differences were considered to be statistically significant when p < 0.05. All of the data are presented as the means ± SEM of three independent experiments.

## Results

### The short form of SIRT3 was downregulated in the murine hypertrophic heart

**Fig 1:**
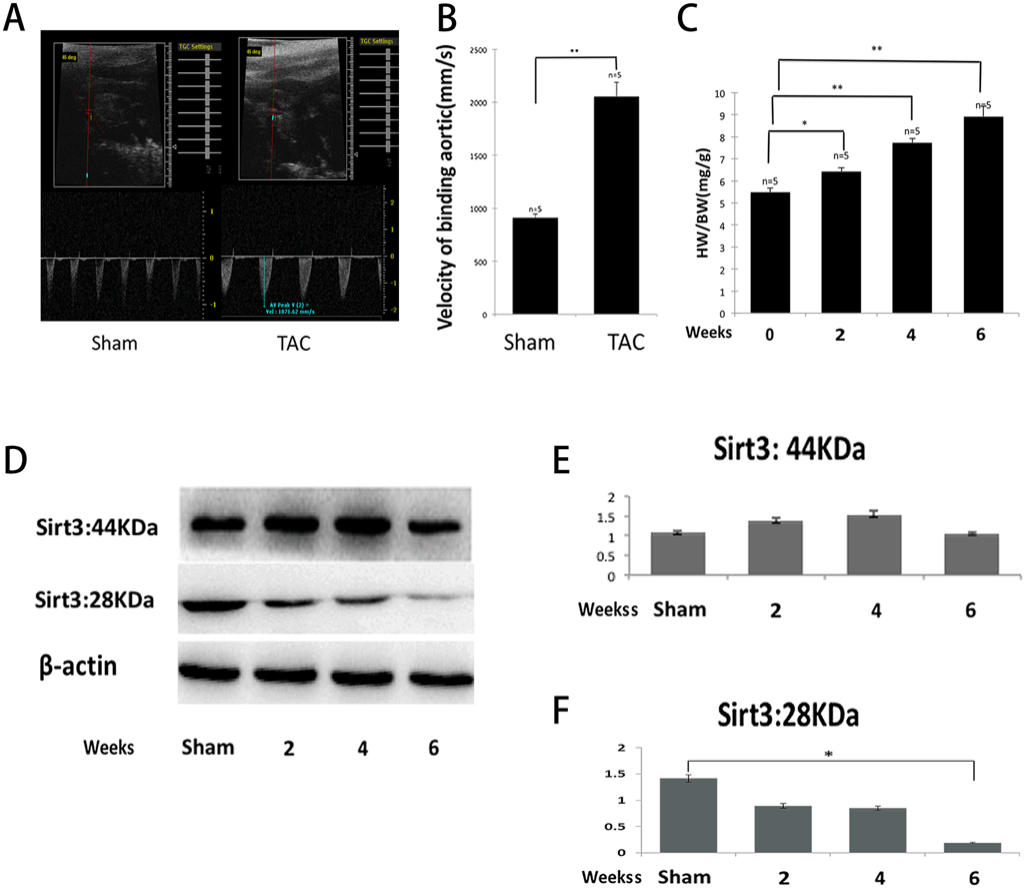
The short form of SIRT3 was downregulated in the murine hypertrophic heart. (A and B) The velocity of the binding aorta was measured by echocardiology. The velocity was significantly elevated one week after TAC. (C) The degree of hypertrophy was assessed by the ratio of the heart weight to the body weight. Mice subjected to TAC produced nearly 60% cardiac hypertrophy at six weeks. (D) The subcellular fractionation from the hearts of WT mice after sham or TAC was analyzed by western blot analysis with antibodies specific for both forms of SIRT3, and β-actin was used as a reference. (E and F) Quantification of both forms of SIRT3 was determined by densitometry analysis, and β-actin was used as a reference. The densitometry value of each band was determined with the ImageJ software. The data are presented as the means ± SEM of three independent experiments. *P<0.05, **P<0.01.

Mouse SIRT3 is a deacetylase that exists ubiquitously in tissues rich in mitochondria: brain, heart, liver, and brown adipose [21]. It is expressed in two forms, a 44-kDa long form and a 28-kDa short form. The long form is localized in the mitochondria, nucleus, and cytoplasm, whereas the short form is localized exclusively in the mitochondria [23,29]. We subjected the mice to TAC for six weeks. To ensure that the aortic binding was definite, we explored the velocity of the binding aorta one week after sham or TAC through echocardiology, and the velocity was significantly elevated after binding (Fig. 1A and 1B). The mice subjected to TAC produced nearly 20%, 40%, and 60% cardiac hypertrophy, respectively, two, four, and six weeks after TAC (Fig. 1C), and we found that the short form of SIRT3 was downregulated after TAC compared with the sham controls (Fig. 1D and 1F), whereas the long form was slightly increased during mild hypertrophy but did not show obvious changes during severe hypertrophy (Fig. 1D and 1E). These results agreed with the findings of a previous study conducted by Sundaresan et al. [21], indicating that only the short form of SIRT3 was consistently downregulated in mice subjected to TAC.

### SIRT3-KO mice had a propensity to develop heart failure in response to TAC

**Fig 2:**
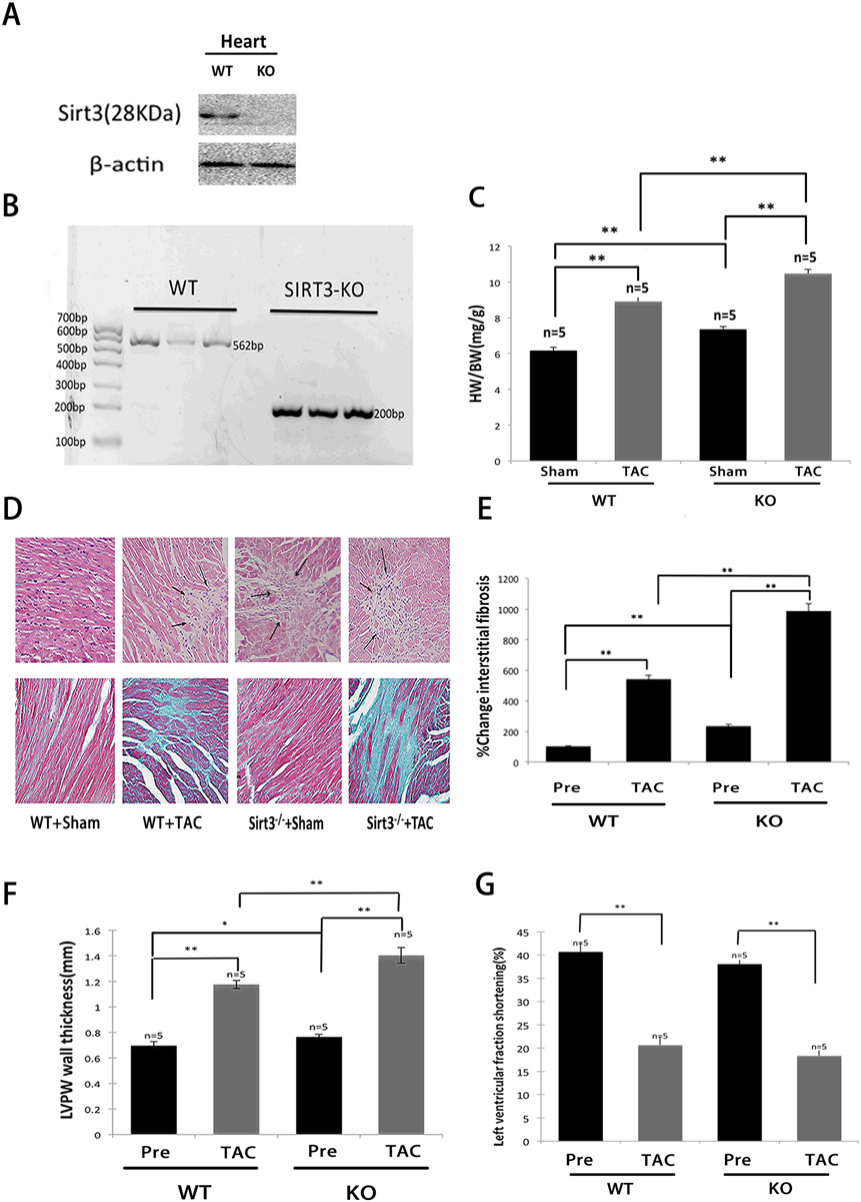
SIRT3-KO mice had a propensity to develop heart failure in response to TAC. (A) SIRT3 protein was absent in the hearts from SIRT3-KO mice. The protein extracts were probed with antibodies to SIRT3 (28 kDa) and β-actin. (B) SIRT3 PCR genotyping. KO, knockout; WT, wild type. (C) The ratio of the heart weight to the body weight was recorded to assess the level of hypertrophy after TAC in SIRT3-KO mice and WT controls. (D) Hematoxylin/eosin-stained cardiac sections from SIRT3-KO mice and WT controls (sham or TAC). The arrows denote areas of cardiomyocyte loss or dropout. Sections of the hearts were stained with Masson’s trichrome to detect fibrosis (blue). (E) Bar graph showing the quantification of fibrosis in the SIRT3-KO mice hearts and WT controls (sham or TAC). (F and G) The LV wall thickness and fractional shortening (FS) were measured with echocardiology as described in the methods section. The data are presented as the means ± SEM of three independent experiments. *P<0.05, **P<0.01.

To further explore the role of SIRT3 in the development of cardiac hypertrophy, we subjected SIRT3-KO mice and their WT controls to TAC. First, immunoblotting with a SIRT3 (28 kDa)-specific antibody showed the absence of SIRT3 protein in the hearts of SIRT3-KO mice (Fig. 2A). The SIRT3 locus was mutated by the deletion of exon 2 and 3, which encode the translational start site and a portion of the catalytic domain [23], and the genotyping of SIRT3-KO mice was confirmed by PCR (Fig. 2B). It has been reported that chronic Angiotensin II infusion (3.0 mg/kg/day for 14 days) produce cardiac hypertrophy and dysfunction in SIRT3-KO mice [21]. In our experiments, we observed similar effects of TAC in these mice, and the ratio of the heart weight to the body weight (HW/BW) in SIRT3-KO mice subjected to TAC was nearly 15% greater than that observed in the WT controls. Under sham conditions, the ratio was also higher in the SIRT3-KO mice (Fig. 2C). The histological examination of cardiac tissue sections from SIRT3-KO mice revealed signs of cellular necrosis in the hearts as shown by areas of cardiomyocyte loss or dropout (Fig. 2D), and higher levels of fibrosis were also observed in the SIRT3-KO mice compared with the WT controls under both sham and TAC conditions (Fig. 2D and 2E). Echocardiology was used to evaluate the cardiac function noninvasively, and the LV wall thickness of the SIRT3-KO mice was greater than that in the WT mice at baseline and was increased to significantly higher levels than those observed in the WT controls after TAC (Fig. 2F). In addition, the quantification of LV fractional shortening showed that the SIRT3-KO mice developed severe contractile dysfunction (Fig. 2G). These findings revealed that the SIRT3-KO mice had a propensity to develop impaired cardiac function and pathogenesis of heart failure, suggesting that SIRT3 may be required to block cardiac hypertrophy.

### SIRT3-KO mice displayed excessive lipid accumulation and decreased palmitate oxidation rates in the heart

**Fig 3:**
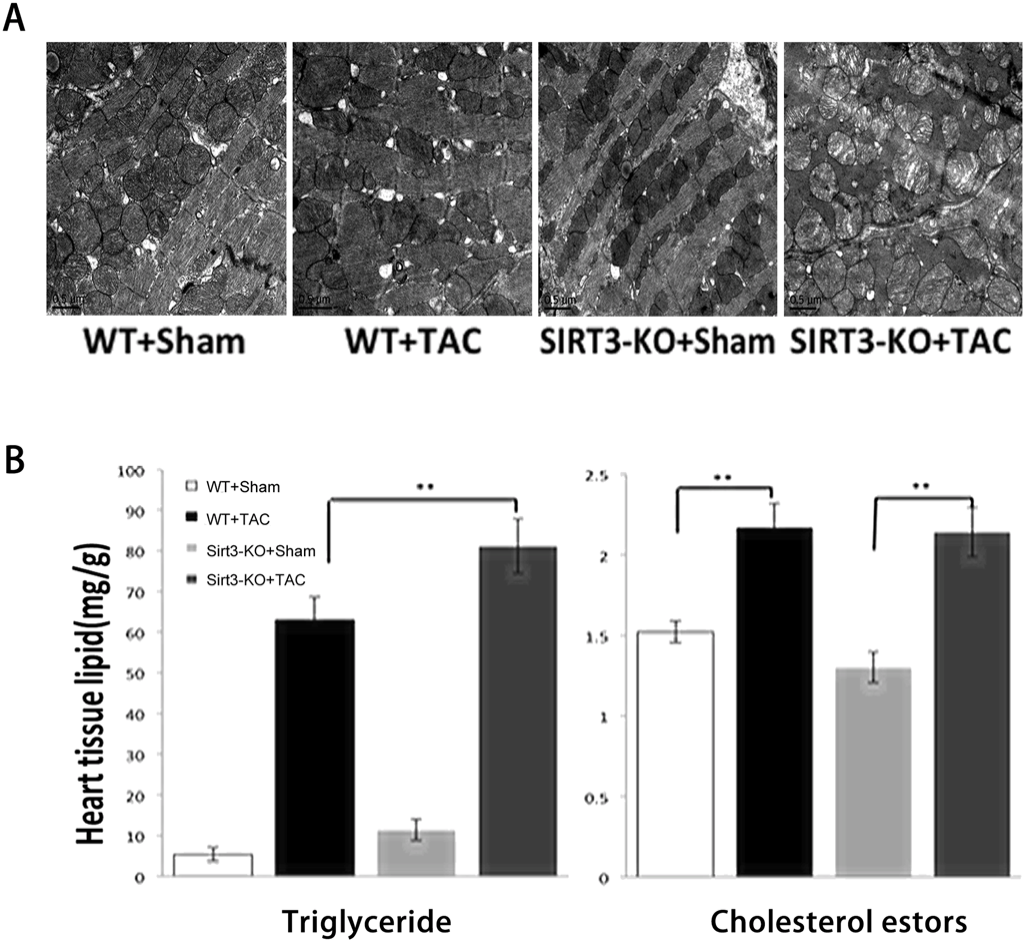
SIRT3-KO mice displayed excessive lipid accumulation and decreased palmitate oxidation rates in the heart. (A) Transmission electron micrographs of cardiac sections from SIRT3-KO mice and WT controls six weeks after sham or TAC. (×20,000). (B) Heart extracts from SIRT3-KO mice and WT controls were analyzed for triglyceride and cholesterol esters. (C) Palmitate oxidation rates in perfused hearts from WT and SIRT3-KO mice after sham or TAC. The data are presented as the means ± SEM of three independent experiments. *P<0.05,**P<0.01.

Cardiac tissue sections from these mice were further examined by transmission electron microscopy, which showed marked changes in the mitochondrial morphology. It appeared that the SIRT3-KO mice did not exhibit any obvious cardiac phenotypes under normal conditions, and the transmission electron micrographs revealed an unusual pattern of lipid body formation in both types of mice after TAC. However, compared with the WT mice, the mitochondria from the transgenic mice became significantly swollen, the mitochondrial cristae were highly disturbed, and large areas of blebbing could be observed in the mitochondria (Fig. 3A). We then used a metabolomics approach to screen multiple metabolic pathways in the heart. The levels of triglyceride and cholesterol were increased in both WT and SIRT3-KO mice subjected to TAC for six weeks (Fig. 3B), and the level of triglyceride was even higher in the SIRT3-KO mice than the controls, which is suggestive of incomplete oxidation of fatty acid, verifying the transmission electron microscopy results. Extensive analyses of the 4-h fasting whole blood and serum showed no difference in the glucose, triglycerides, and cholesterol levels between the SIRT3-KO and WT mice (S1 Table). To directly assess fatty acid oxidation, the degree of *ex vivo* palmitate oxidation was measured in the hearts from WT and SIRT3-KO mice. In the WT mice, the palmitate oxidation rates were 30% lower in the hypertrophic hearts compared with the sham controls, and in the SIRT3-KO mice, the palmitate oxidation rates were significantly lower under both sham and TAC conditions (Fig. 3C). Recent studies conducted with SIRT3-KO mice revealed the important role of SIRT3 in regulating free fatty acid metabolism under stress. Hirschey et al. observed that the SIRT3-KO mice presented a 50% reduction in free fatty acid oxidation, which resulted in the accumulation of long-chain fatty acids in the liver upon fasting [14]. The defect in free fatty acid oxidation was correlated with increased acetylation of long-chain acyl-CoA dehydrogenase (LCAD) in SIRT3-deficient mice, which led a 40% decrease in the enzymatic activity of LCAD. Cardiac hypertrophy is also associated with reduced free fatty acid oxidation and the SIRT3 levels are reduced in hypertrophic/failing hearts [21]. Collectively, these results revealed that SIRT3-KO mice subjected to hypertrophic stimuli displayed excessive lipid accumulation and decreased fatty acid oxidation rates in the heart, which implied that similar SIRT3-mediated mechanisms can operate in the heart to regulate free fatty acid oxidation as it does in the liver.

### SIRT3 controlled the acetylation status of LCAD *in vivo* and *in vitro*

**Fig 4:**
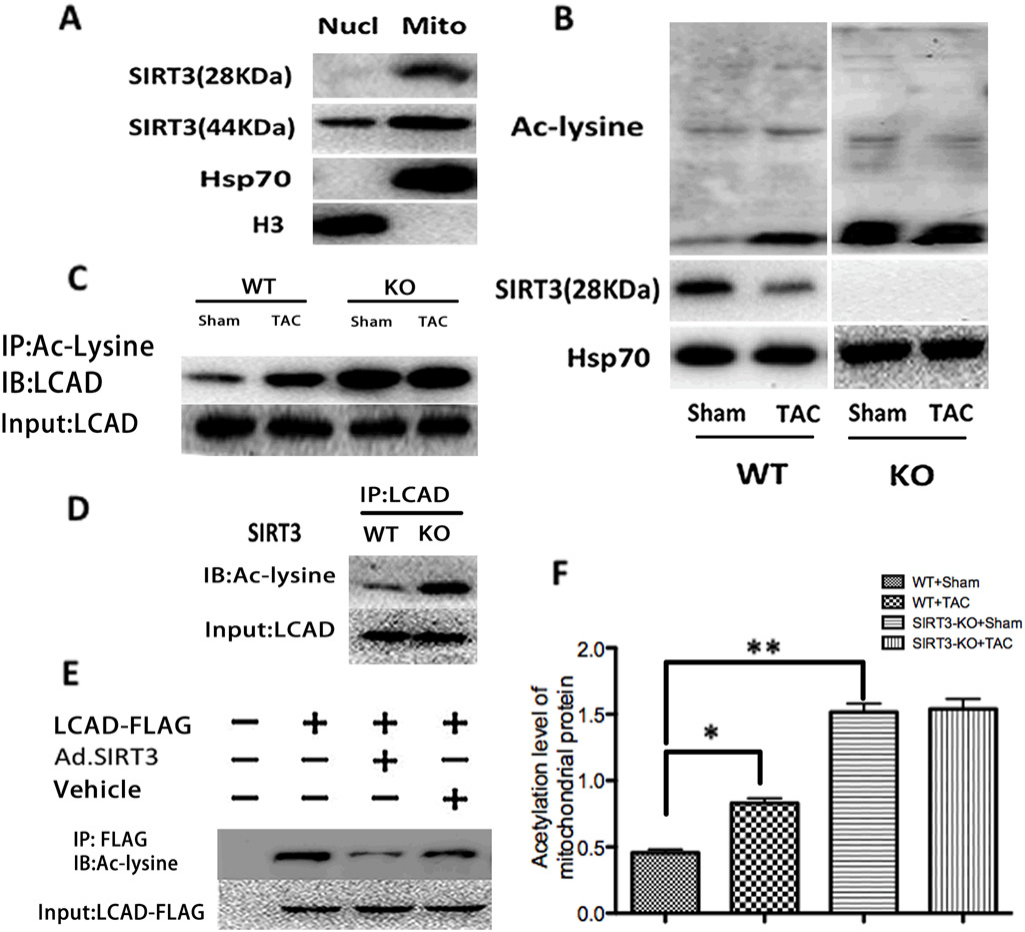
SIRT3 controlled the acetylation status of LCAD *in vivo* and *in vitro*. (A) The short form of SIRT3 (28 kDa) was localized exclusively in the mitochondria. Nuclear and mitochondrial protein extracts were immunoblotted with antibodies against the indicated proteins such as histone 3 and Hsp70. (B) The global mitochondrial acetylation level was assessed in both WT and SIRT3-KO mice subjected to sham or TAC. (C) Mitochondria extracts from SIRT3-KO mice and WT controls (sham or TAC) were immunoprecipitated with Ac-Lysine antiserum and analyzed with anti-LCAD. (D) LCAD immune complexes from hearts of SIRT3-KO mice and WT controls were immunoblotted with Ac-Lysine antibodies, and probing with LCAD antibodies revealed the total LCAD levels. (E) Expression vectors encoding FLAG-tagged murine LCAD were cotransfected with an expression vector for either Ad.SIRT3 or vehicle into cardiomyocytes. The acetylation levels for murine LCAD were measured after immunoprecipitation (anti-FLAG) by western blotting with Ac-Lysine antiserum. (F) Quantification of the acetylation level of mitochondrial proteins in WT and SIRT3-KO mice. The data are presented as the means ± SEM of three independent experiments. *P<0.05, **P<0.01.

To further define the mechanism through which SIRT3-KO mice subjected to TAC showed excessive lipids accumulation in the heart, we explored the global mitochondrial acetylation status in both WT and SIRT3-KO mice. First, the heart nucleus and mitochondria were purified according to standard procedures, and the nuclear and mitochondrial protein extracts were analyzed by immunoblotting using antibodies against both isoforms of SIRT3 and organelle marker proteins (Fig. 4A). In our experiments, we observed that full-length SIRT3 was localized in both the nucleus and mitochondria, whereas the short form of SIRT3 was localized exclusively in the mitochondria, which is in agreement with previous reports [23,29]. Because the enzymes involved in fatty acid oxidation are localized in the mitochondria, we intended to explore whether the short form of SIRT3 (28 kDa) participates in the regulation of cardiac fatty acid oxidation. The results showed obvious hyperacetylation of the mitochondrial proteins in SIRT3-KO mice, and in WT mice, a relative increase in the acetylation level of mitochondrial proteins was accompanied by a parallel decrease in the short form of SIRT3 (28 kDa) (Fig. 4B and 4F). Because most intermediate metabolic enzymes are acetylated and acetylation can directly affect the enzyme activity or stability [30], we assessed the acetylation level of long-chain acyl CoA dehydrogenase (LCAD), a key enzyme involved in fatty acid oxidation. The endogenous mitochondrial proteins were immunoprecipitated with anti-acetyllysine antiserum and were analyzed by western blotting using an antibody specific to LCAD. This experiment demonstrated that LCAD was acetylated under normal conditions and became hyperacetylated during hypertrophy (Fig. 4C), and when the same experiment was conducted using mitochondria extracted from SIRT3-KO mice, LCAD was profoundly hyperacetylated under both normal state and hypertrophy (Fig. 4C). This result demonstrated that SIRT3 was necessary for LCAD deacetylation. To further confirm this observation, we immunoprecipitated LCAD from mitochondrial extracts under normal states and analyzed the immune complexes with antibodies to acetyllysine. LCAD from SIRT3-KO mice exhibited higher levels of acetylation than that from WT mice, confirming LCAD hyperacetylation in SIRT3 deficiency (Fig. 4D). To test the ability of SIRT3 to directly deacetylate LCAD, expression vectors encoding FLAG-tagged murine LCAD were cotransfected with an expression vector for SIRT3 or vehicle into cardiomyocytes. The acetylation levels for murine LCAD were measured by western blotting with an antibody for acetyllysine after immunoprecipitation with anti-FLAG antiserum (Fig. 4E). This experiment demonstrated that the overexpression of SIRT3 could deacetylate LCAD directly. LCAD lysine 42 was previously proven to be a critical lysine residue for the regulation of LCAD enzymatic activity, and hyperacetylated LCAD showed a nearly 40% reduction in enzymatic activity in the liver [14]. Together, these observations showed that SIRT3 may be able to regulate fatty acid oxidation through the deacetylation of LCAD in the heart.

### Overexpression of SIRT3 attenuated lipid accumulation *in vitro* and blocked the cardiac hypertrophic response

**Fig 5:**
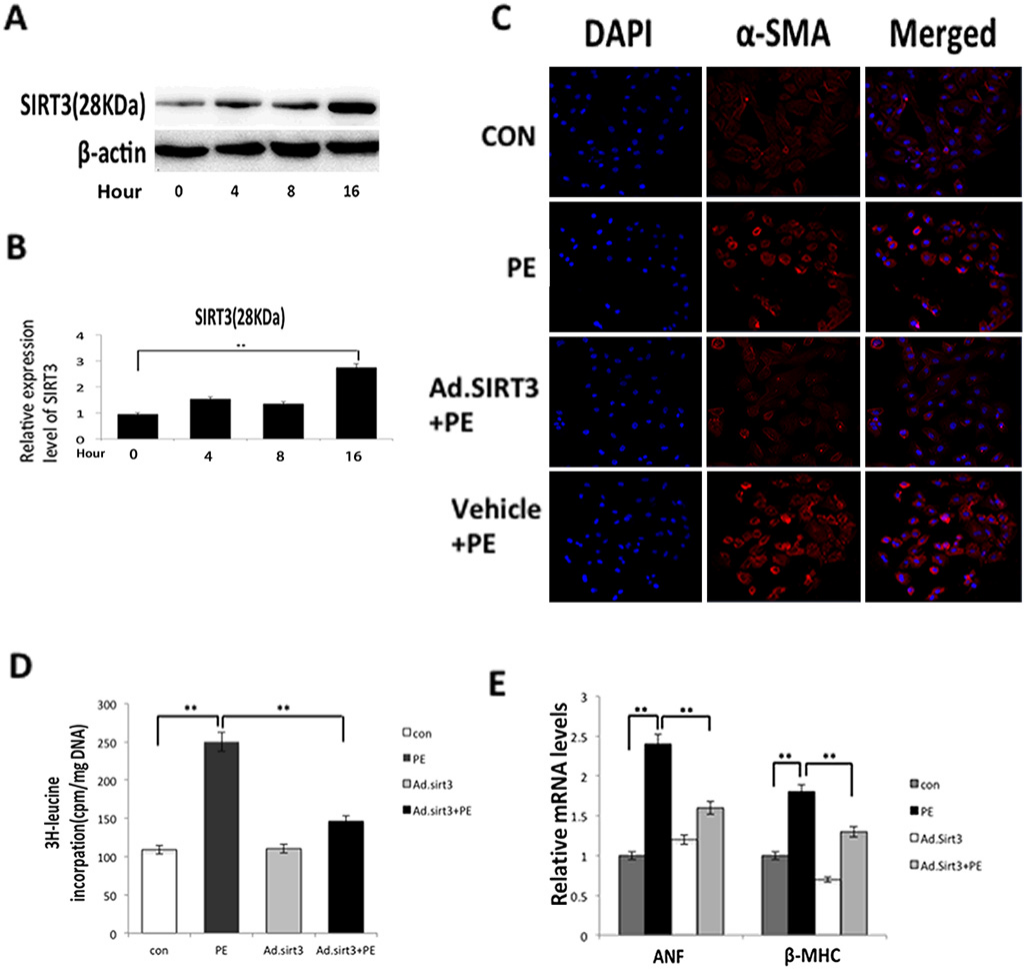
Overexpression of SIRT3 blocked the cardiac hypertrophic response *in vitro*. (A) Expression level of SIRT3 in cardiomyocytes after infection with Ad.SIRT3 or vehicle at an MOI of 10 for 16 h. (B) Quantification of SIRT3 at different time points after infection with Ad.SIRT3. (C) Cardiomyocytes were infected with Ad.SIRT3 or vehicle and then treated with PE (20 μM) or blank control (saline). The cells were stained for α-SMA (red), and the nucleus positions were determined by DAPI staining (blue). (D) The incorporation of [^3^H]-leucine into total cellular proteins was determined and normalized to the DNA content of the cells. (E) mRNA levels of the indicated fetal genes (*anf* and *myh-7*) in cardiomyocytes infected with Ad.SIRT3 and then treated or not treated with PE. The data are presented as the means ± SEM of three independent experiments. *P<0.05, **P<0.01.

**Fig 6:**
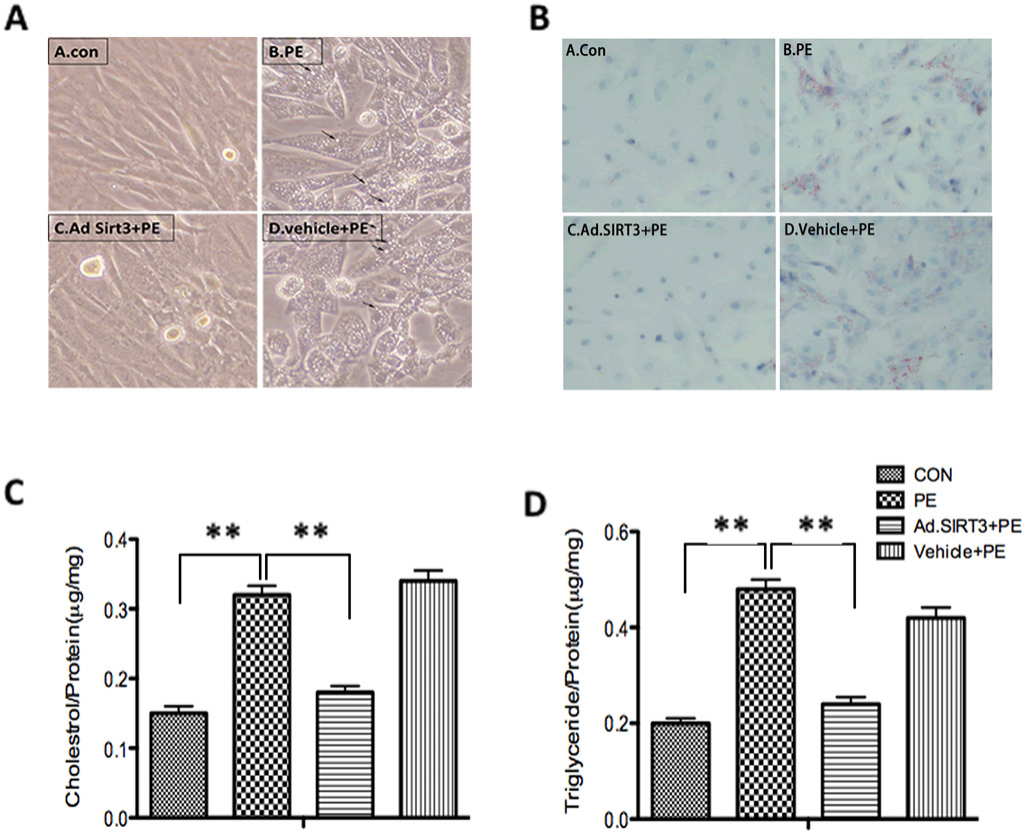
Overexpression of SIRT3 attenuated lipid accumulation *in vitro*. (A and B) Cardiomyocytes were infected with Ad.SIRT3 or vehicle and then treated with PE or blank control. The cells were observed using an optical microscope and stained or not stained with oil red. (C) The cholesterol level in cardiomyocytes was measured in response to PE. (D) The triglyceride level in cardiomyocytes was measured in response to PE. The data are presented as the means ± SEM of three independent experiments. *P<0.05, **P<0.01.

The cardiomyocytes that were infected with Ad.SIRT3 at an MOI of 10 for 16 h presented a three-fold higher level of SIRT3 than the controls (Fig. 5A and 5B). According to the above-described *in vitro* experiment that revealed the overexpression of SIRT3 deacetylates LCAD (Fig. 4D), we used phenylephrine (PE) (20 μM) or blank control (saline) to treat cardiomyocytes that were infected with Ad.SIRT3 or vehicle for 48 h. An immunofluorescence analysis revealed that α-SMA, a major marker for cardiomyocyte hypertrophy, is strongly expressed in response to PE, whereas the overexpression of SIRT3 reduced the expression of α-SMA in cardiomyocytes (Fig. 5C). The PE-induced protein synthesis was measured by [^3^H]-leucine incorporation into the total cellular protein (Fig. 5D), which showed that the induction of protein synthesis was inhibited by the overexpression of SIRT3, and the analysis of the ANF and β-MHC mRNA levels also demonstrated that the overexpression of SIRT3 could block the activation of fetal gene expression induced by PE stimuli (Fig. 5E). These results agreed with those found in a previous study conducted by Sundaresan et al. [21]. In addition, using an optical microscope, some droplets were observed in the cytosol of the cells in response to PE stimuli, and the cardiomyocytes of different groups were then stained with oil red, which could detect lipid accumulation in the cells. It was shown that the overexpression of SIRT3 attenuated lipid accumulation in response to PE stimuli (Fig. 6A and 6B). We also determined the intracellular accumulation of cholesterol and triglyceride in cardiomyocytes to confirm the apparent increase in the lipid content in response to PE stimuli. In the cells pretreated with Ad.SIRT3 infection compared with the controls, the cholesterol and triglyceride accumulation was reduced 40% and 50% respectively (Fig. 6C and 6D). We observed that the overexpression of SIRT3 was able to attenuate lipid accumulation in cardiomyocytes. These findings revealed that SIRT3 was able to protect cardiomyocytes from hypertrophic stimuli by attenuating lipid accumulation.

## Discussion

This study was designed to investigate the role of SIRT3 in cardiac hypertrophy-related lipid accumulation. It was shown that the short form of SIRT3 was downregulated during hypertrophy in WT mice, and this effect was accompanied by a higher acetylation level of LCAD. Compared with WT mice, the LVPW, HW/BW and cardiac fibrosis ratio were higher in SIRT3-KO mice under both sham and TAC conditions, and when subjected to TAC, more lipids accumulated in the SIRT3-KO mice hearts. In addition, the LVPW and HW/BW increased with aging, implying that SIRT3-KO mice could have spontaneous cardiac hypertrophy (S3A-B Fig.). All of these results indicated that the SIRT3-KO mice were inclined to develop heart failure and lipid metabolism disorders. Further investigation of the underlying mechanism demonstrated that the overexpression of SIRT3 in cardiomyocytes could attenuate lipid accumulation through the deacetylation of LCAD, which participates in fatty acid oxidation. Our findings suggested that SIRT3 may block cardiac hypertrophy by inhibiting lipid metabolism disorders and attenuate lipid accumulation in the mitochondria through the deacetylation of LCAD.

In the TAC-induced mouse hypertrophy model, we found that the short form of SIRT3 was downregulated during hypertrophy and that this effect was associated with a higher global mitochondrial acetylation status. However, the mechanism underlying the changes in the expression of SIRT3 during hypertrophy has not been examined. Based on studies of other sirtuins whose levels increased during mild stress conditions, such as CR [31], it can be presumed that a change in the cellular NAD/NADH ratio following stress may, in part, contribute to the change in the levels of SIRT3 [32]. In severe pathologic hypertrophy and heart failure, the overactivation of poly (ADPribose) polymerase-1 increased the consumption of NAD [33]. The short form of SIRT3 may be downregulated as a result of a low NAD level in hypertrophy. In our study, the short form of SIRT3 was downregulated consistently after TAC for six weeks, whereas the long form was increased slightly during mild hypertrophy but did not show obvious changes during severe hypertrophy. In a previous study conducted by Sundaresan et al. [22], both forms of SIRT3 were increased during mild hypertrophy, and the short form of SIRT3 was downregulated during severe hypertrophy of the heart. Thus, we verified the expression of SIRT3 during hypertrophy with another specific antibody that can detect all forms of SIRT3 (S4 Fig.). Although we detected two additional bands (36–37 kDa) of SIRT3, we obtained similar results as those presented in Fig. 1D. However, the changing trend may not arouse controversy regarding the function of SIRT3. The long form of SIRT3, which localizes in the mitochondria, cytoplasm, and nucleus, has been proven to interact with various transcription factors, such as Ku70 and foxo3a, and protects cardiomyocytes by promoting the antioxidant defense mechanisms of cells.

Hypertrophy, in the context of hypertension, is usually interpreted primarily as an adaptive response in an effort to normalize mechanical wall stress. More recently, the importance of the metabolic remodeling processes inherent in the hypertrophic growth response (whether primary or secondary) has been recognized [34,35]. Altered energy demands, shifts in substrate utilization and increased oxidative stress are observed in the hypertrophic heart. Although endocrinological disturbances (such as insulin resistance) induce alterations in substrate availability, the extent to which metabolic remodeling in the heart drives the hypertrophic process is not yet clear. Increased production of reactive oxygen species (ROS) and disturbed Ca^2+^ handling are both implicated as causative molecular signals mediating the link between altered substrate utilization and hypertrophic growth responses [36]. Thus, understanding the mechanism of lipid metabolism disorders may help prevent hypertrophic growth response. Our data showed that, compared with wild-type mice, SIRT3-KO mice subjected to TAC had a propensity to develop heart failure and lipid accumulation in the mitochondria. This finding implies that SIRT3 may play an essential role in the regulation of lipid metabolism in the mitochondria.

In addition, our data showed that severe lipid metabolism disorders are observed in the hypertrophic heart, and we tested some factors involved in mitochondria fatty acid oxidation. As detailed in a previous report, PGC-1α controls SIRT3 gene expression, and SIRT3 is essential for the PGC-1α-dependent induction of ROS-detoxifying enzymes and several components of the respiratory chain [37,38]. In addition, PGC-1α and PPARα cooperate to activate genes encoding enzymes involved in cardiac fatty oxidation [39]. Thus, we tested the PGC-1α and PPARα levels in WT and SIRT3-KO mice and found that the PGC-1α and PPARα levels were decreased in response to TAC but showed no differences between WT and SIRT3-KO mice under either normal or disease conditions (S1 Fig.). Collectively, these results implied that PGC-1α and PPARα may be upstream of SIRT3, whereas SIRT3 may be essential for the PGC-1α- and PPARα-dependent regulation of fatty acid oxidation. Along with these transcription factors, we also observed decreased mRNA levels of CPT-1 and LCAD, which are key enzymes in fatty acid import and oxidation (S2 Fig.). LCAD lysine 42 has been proven to be a critical lysine residue for the regulation of LCAD enzymatic activity, and hyperacetylated LCAD showed a nearly 40% reduction in enzymatic activity in the liver [14]. Thus, we want to explore whether SIRT3 regulates lipid metabolism through the deacetylation of LCAD, and further studies on the role of the SIRT3-induced regulation of other enzymes involved in fatty acid import or mobilization are required.

We also found that the decrease in the expression of the short form of SIRT3 during hypertrophy was concomitant with a relatively higher acetylation level of cardiomyocyte proteins. More recent proteomic analyses of lysine-acetylated mitochondrial proteins showed that as much as 50% of all mitochondrial proteins are acetylated and that proteins involved in energy metabolism are overrepresented. This includes proteins associated with the tricarboxylic acid (TCA) cycle, oxidative phosphorylation, β-oxidation of lipids, amino acid metabolism, carbohydrate metabolism, nucleotide metabolism, and the urea cycle [30]. Combined with the results observed in SIRT3-deficient mice, we speculated that lipid metabolism disorders during hypertrophy are due to a loss of deacetylation function caused by a lack of SIRT3.

Most intermediate metabolic enzymes are acetylated, and acetylation can directly affect the enzyme activity or stability. In addition, the acetylation of metabolic enzymes changed in response to alterations in the extracellular nutrient availability, providing evidence for a physiological role of dynamic acetylation in metabolic regulation. Of the many key enzymes involved in fatty acid oxidation, in our study, we showed that LCAD was hyperacetylated during hypertrophy, and this effect was even more profound in SIRT3-deficient mice, in which LCAD was relatively hyperacetylated in both the normal state and hypertrophy. Previous studies have found that LCAD lysine 42 is a critical lysine residue for the regulation of LCAD enzymatic activity and that hyperacetylated LCAD shows ~40% reduced enzymatic activity in the liver [14]. These findings revealed that the overexpression of SIRT3 may activate LCAD through deacetylation.

In our experiments, the mitochondria from SIRT3-KO mice were morphologically similar to the WT mitochondria, as observed by electron microscopy, and the levels of cholesterol and triglycerides were comparable under normal conditions between SIRT3-KO and WT mice. However, hyperacetylation of LCAD was observed in SIRT3-KO mice under normal conditions. According to a previous study, which focused on the role of SIRT3 in the regulation of fatty acid oxidation in the liver, WT and SIRT3-KO tissue homogenates showed equal abilities to oxidize palmitate under low substrate concentrations. However, as the lipid concentrations increased, the liver tissue from fasted SIRT3-KO mice exhibited a lower oxidizing capacity than WT tissue [14]. Cardiomyocyte hypertrophy is characterized by an increase in cell size, enhanced protein synthesis, and heightened organization of the sarcomere at the cellular level, indicating the need of importing a greater amount of fatty acids into cardiomyocytes as energy [40]. Thus, under normal conditions, the lower concentration of fatty acid may not exceed the FAO capacity and arouse lipid metabolism disorders, even in the SIRT3-KO mice. However, during hypertrophy, excessive fatty acids were imported into cardiomyocytes and induced significant lipid accumulation, which may be, in part, due to hyperacetylation and reduced LCAD activity. In addition, our data showed that the overexpression of SIRT3 in cardiomyocytes could attenuate lipid accumulation in hypertrophy induced by PE stimulation. SIRT3 is a longevity factor and the only member of the sirtuin family whose increased expression has been linked to an extended life span in humans. Our findings suggested that SIRT3 may block heart hypertrophy by inhibiting lipid metabolism disorders and may attenuate lipid accumulation in mitochondria through the deacetylation of LCAD and that the manipulation of SIRT3 expression may provide a new approach to combat hypertrophy.

## Supporting Information

S1 Fig The cardiac PGC-1α and PPARα protein levels. The cardiac PGC-1α and PPARα levels in WT and SIRT3-KO mice under sham or TAC conditions were determined by western blot.
(TIF)

S2 Fig The cardiac mRNA levels of CPT-1 and LCAD. The cardiac mRNA levels of CPT-1 and LCAD in WT and SIRT3-KO mice under sham and TAC conditions were determined by Q-PCR. The data are presented as the means ± SEM of three independent experiments. *P<0.05.
(TIF)

S3 Fig LVPW (mm) and HW/BW (mg/g) statistics in SIRT3-KO mice (aged from 8 to 32 weeks). (A) The LV posterior wall thickness was measured with echocardiology. (B) The degree of hypertrophy was assessed by the ratio of the heart weight to the body weight. The data are presented as the means ± SEM of three independent experiments. *P<0.05, **P<0.01.
(TIF)

S4 Fig The cardiac SIRT3 protein levels. The levels of all forms of SIRT3 during cardiac hypertrophy were determined by western blot.
(TIF)

S1 Table Serum chemistry of WT and SIRT3-KO mice subjected to sham or TAC. None of the data were significantly different between WT and SIRT3-KO mice. For serum chemistry, retro-orbital blood was collected after 4-h fasting under isoflurane (2–2.5%) anesthesia. The 4-h fasting glucose levels were measured with tail bleed without anesthesia. The data are presented as the means ± SEM of three independent experiments. *P<0.05, **P<0.01.
(DOCX)
