# Correction: Mouse SIRT3 Attenuates Hypertrophy-Related Lipid Accumulation in the Heart through the Deacetylation of LCAD

**DOI:** 10.1371/journal.pone.0155173

**Published:** 2016-05-04

**Authors:** Tongshuai Chen, Junni Liu, Na Li, Shujian Wang, Hui Liu, Jingyuan Li, Yun Zhang, Peili Bu

Fig 3 appears incorrectly in the published article. Please see the correct Fig 3 and its caption here.

**Fig 3:**
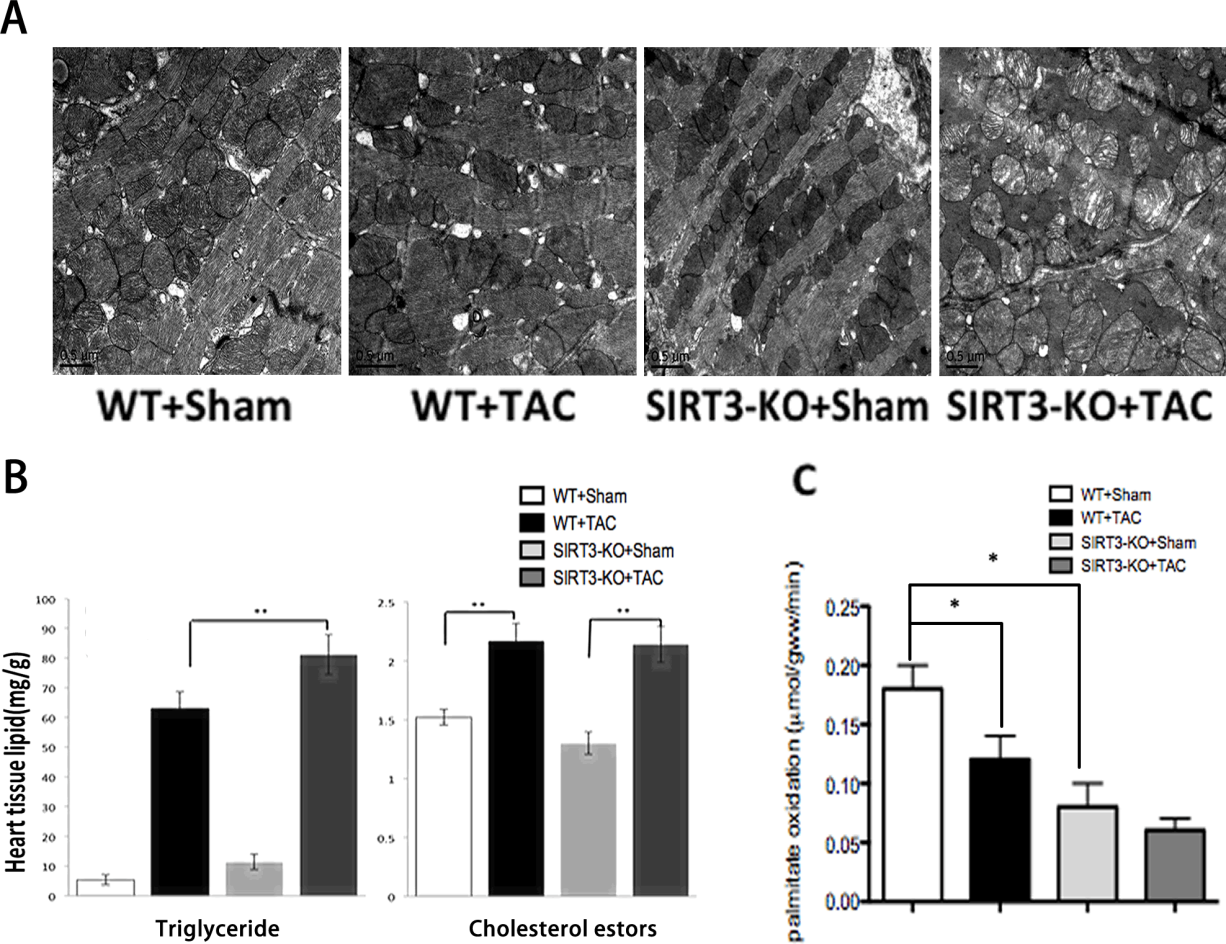
SIRT3-KO mice displayed excessive lipid accumulation and decreased palmitate oxidation rates in the heart. (A) Transmission electron micrographs of cardiac sections from SIRT3-KO mice and WT controls six weeks after sham or TAC. (×20,000). (B) Heart extracts from SIRT3-KO mice and WT controls were analyzed for triglyceride and cholesterol esters. (C) Palmitate oxidation rates in perfused hearts from WT and SIRT3-KO mice after sham or TAC. The data are presented as the means ± SEM of three independent experiments. *P<0.05,**P<0.01.
