# Supplementary figures and images for: Mouse SIRT3 Attenuates Hypertrophy-Related Lipid Accumulation in the Heart through the Deacetylation of LCAD
Source: PLoS One. 2015 Mar 6;10(3):e0118909. doi: 10.1371/journal.pone.0118909 (PMC4351969; doi:10.1371/journal.pone.0118909)

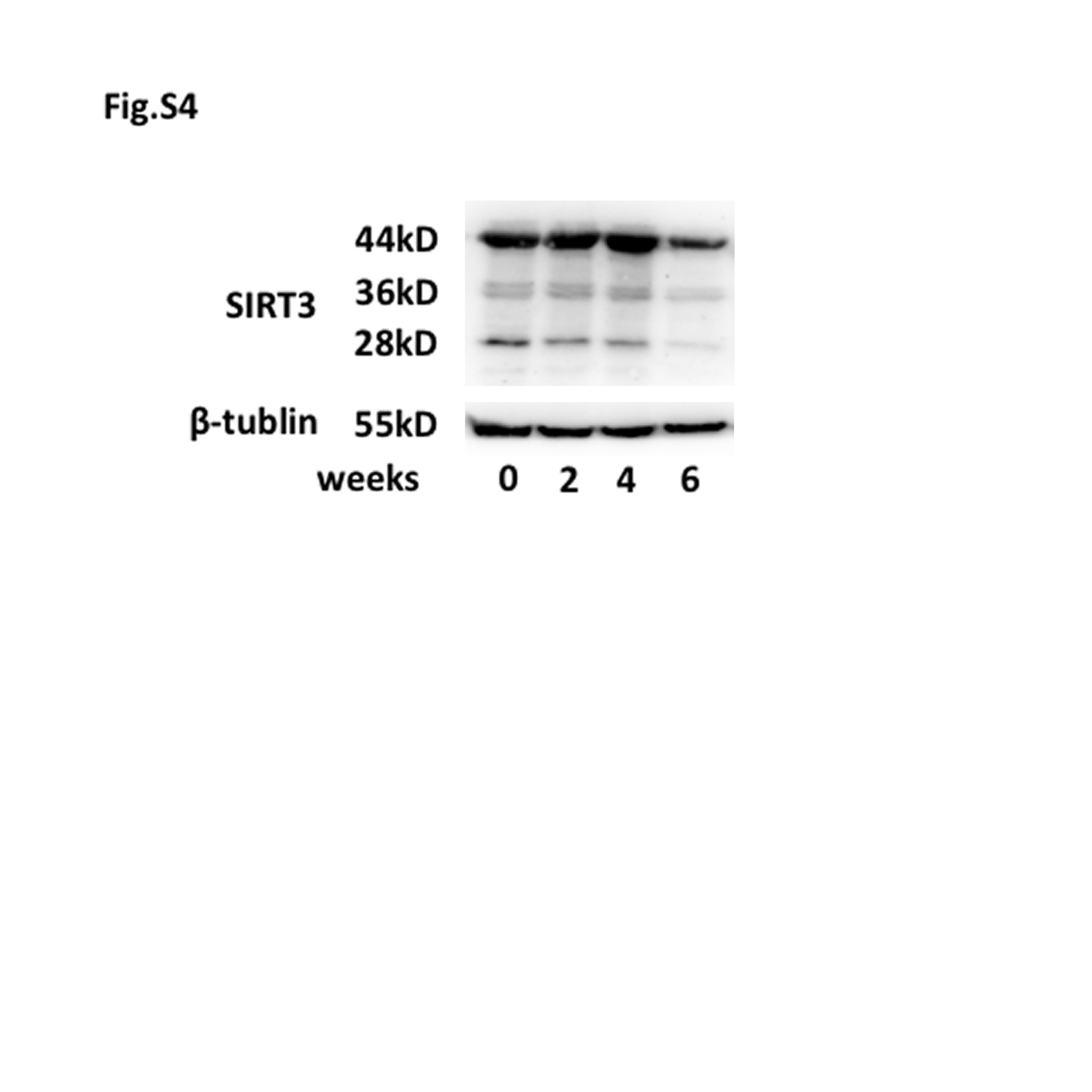

Supplement: S4 Fig — The levels of all forms of SIRT3 during cardiac hypertrophy were determined by western blot. (TIF) [file pone.0118909.s004.tif]
