# Supplementary material for: Mouse SIRT3 Attenuates Hypertrophy-Related Lipid Accumulation in the Heart through the Deacetylation of LCAD
Source: PLoS One. 2015 Mar 6;10(3):e0118909. doi: 10.1371/journal.pone.0118909 (PMC4351969; doi:10.1371/journal.pone.0118909)
Supplement: S1 Table — None of the data were significantly different between WT and SIRT3-KO mice. For serum chemistry, retro-orbital blood was collected after 4-h fasting under isoflurane (2–2.5%) anesthesia. The 4-h fasting glucose levels were measured with tail bleed without anesthesia. The data are presented as the means ± SEM of three independent experiments. *P<0.05, **P<0.01. (DOCX) [file pone.0118909.s005.docx]

**Table S1. Serum chemistry for WT and SIRT3-KO mice subjected to sham or TAC.**

| Groups | WT+Sham | WT+TAC | SIRT3-KO+Sham | SIRT3-KO+TAC |
| --- | --- | --- | --- | --- |
| *n* | 5 | 5 | 5 | 5 |
| Glucose (mg/dl) | 140±9 | 135±8 | 142±7 | 137±6 |
| Triglycerides (mg/dl) | 75±4 | 78±5 | 72±4 | 76±5 |
| Cholesterol (mg/dl) | 82±4 | 80±3 | 84±5 | 81±4 |

None of the data were significantly different between WT and SIRT3-KO mice. For serum chemistry, retro-orbital blood was collected after 4-h fasting under isoflurane (2–2.5%) anesthesia. The 4-h fasting glucose levels were measured with tail bleed without anesthesia. The data are presented as the means ± SEM of three independent experiments. * *P*<0.05, ***P*<0.01.
